# Implementing a Statistical Parametric Speech Synthesis System for a Patient with Laryngeal Cancer

**DOI:** 10.3390/s22093188

**Published:** 2022-04-21

**Authors:** Krzysztof Szklanny, Jakub Lachowicz

**Affiliations:** Multimedia Department, Polish-Japanese Academy of Information Technology, 02-008 Warsaw, Poland; s12054@pjwstk.edu.pl

**Keywords:** speech synthesis, parametrical synthesis, deep neural networks, laryngeal cancer

## Abstract

Total laryngectomy, i.e., the surgical removal of the larynx, has a profound influence on a patient’s quality of life. The procedure results in a loss of natural voice, which in effect constitutes a significant socio-psychological problem for the patient. The main aim of the study was to develop a statistical parametric speech synthesis system for a patient with laryngeal cancer, on the basis of the patient’s speech samples recorded shortly before the surgery and to check if it was possible to generate speech quality close to that of the original recordings. The recording made use of a representative corpus of the Polish language, consisting of 2150 sentences. The recorded voice proved to indicate dysphonia, which was confirmed by the auditory-perceptual RBH scale (roughness, breathiness, hoarseness) and by acoustical analysis using AVQI (The Acoustic Voice Quality Index). The speech synthesis model was trained using the Merlin repository. Twenty-five experts participated in the MUSHRA listening tests, rating the synthetic voice at 69.4 in terms of the professional voice-over talent recording, on a 0–100 scale, which is a very good result. The authors compared the quality of the synthetic voice to another model of synthetic speech trained with the same corpus, but where a voice-over talent provided the recorded speech samples. The same experts rated the voice at 63.63, which means the patient’s synthetic voice with laryngeal cancer obtained a higher score than that of the talent-voice recordings. As such, the method enabled for the creation of a statistical parametric speech synthesizer for patients awaiting total laryngectomy. As a result, the solution would improve the quality of life as well as better mental wellbeing of the patient.

## 1. Introduction

The larynx is the most common localization of malignant head and neck cancers. In Poland, laryngeal cancer accounts for 2.3% of all cancers in men and 0.5% cancers in women [1,2,3,4]. Symptoms of laryngeal cancer include persistent hoarseness, globus sensation, a sore throat, an earache, a cough or weight loss. The risk factors include alcohol consumption, smoking, HPV-16 infection, reflux and exposure to toxic fumes of nickel compounds, sulfuric acid, asbestos or heavy metals [5,6,7]. HPV-16 (human papilloma virus) infection can lead to uncontrolled cell divisions of the cervical epithelium, which can end in cervical cancer [8,9]. In its initial stage, laryngeal cancer may not display clear symptoms, which can lead to a late diagnosis and, consequently, to a more aggressive treatment: surgery and/or chemotherapy and/or radiotherapy [1,6,9].

While early, locally advanced cancer can be treated effectively, for instance by means of microsurgery, but more advanced laryngeal cancer may require a complete removal of the larynx (total laryngectomy) [9]. This will always have a profound impact on the patient’s quality of life, as the loss of natural voice constitutes a significant socio-psychological problem for patients. Regrettably, in many cases, this often leads to a patient’s social isolation and depression [9,10,11,12].

There are three methods of voice restoration following laryngectomy [13]. The first involves the implantation of an artificial larynx. Thanks to the implant, the air can be directed from the lungs to the esophagus in order to create the primary laryngeal tone [14]. In order to be able to speak, the patient has to close off the trach tube opening, which is a major inconvenience. However, patients recover their voice fairly quickly, usually within several days. This kind of speech is known as tracheoesophageal speech (TE) [15,16]. Another method of voice recovery involves the learning of esophageal speech (ES) [17]. It requires the patient to learn to burp out the air returning from the stomach or esophagus. This is far more difficult to learn, while patients often feel uneasy about burping, as it is thought to be rude. Statistically, 40% of all patients manage to master this method, but merely 15% of them actually make use of it [11,18]. The third method involves the use of an electrolarynx [19], a device that generates the fundamental frequency when held against the neck. The generated voice sounds artificial and flat, similar in quality to that of formant synthesis (defined below).

Clearly then, there is a need to create augmentative and alternative communication methods, allowing those who cannot produce speech, or have a limited ability to produce speech, to communicate. These include sign language as well as voice output communication aids (VOCAs) [20]. There are several types of speech synthesis used in the VOCA systems, such as formant synthesis, concatenation synthesis, unit selection speech synthesis, and statistical parametric speech synthesis based on the hidden Markov model [21].

The concept of a digital formant speech synthesizer was introduced by Dennis Klatt in 1979 [22]. This kind of synthesis involves using cascade and/or parallel digital filters to model the vocal tract transfer function in the frequency domain. The sound generated in this way has a characteristic tone quality, reproducing the typical formants of speech sounds. Generating intelligible speech requires the reproduction of three formants. Five formants make it possible to generate speech of sufficiently high quality. Each of the formants is modelled with a formant frequency and a resonance band [23].

Models for concatenative speech synthesis, developed since the 1970s, have gained considerable popularity due to their ability to generate high-quality natural-sounding speech. In concatenative synthesis, speech is generated by concatenating acoustic segments, such as phones, diphones, triphones and syllables [24]. Thanks to its sound-to-sound transition characteristic, the diphone is the most common unit which ensures high-quality natural speech. The small size of its database is an advantage of this type of synthesis. The smaller the database, the better, as speech will be generated more quickly, and the hardware requirements will be less demanding [25]. 

Rather than having a database containing a single occurrence of a given sound unit, unit selection (corpus-based) speech synthesis relies on a special corpus that comprises a number of its occurrences in different contexts, making use of units of varying duration. Owing to this, it is often possible to avoid artificial concatenation points, allowing for more natural-sounding speech [26]. The most important element responsible for the acoustic segment selection is the cost function. It consists of target cost and a concatenation cost (joint cost). The concatenation cost is used to assess the degree to which two units match if they are not in adjacent positions in the acoustic database. The unit selection cost searches out units that will most closely match the linguistic features of the target sentence [27,28].

The HMM-based speech synthesis system (HSS) utilizes the hidden Markov models (HMMs) [28]. In a way, it is similar to concatenation. However, in this case, instead of using segments of natural speech, the synthesis process relies on context-dependent HMMs. These models are concatenated according to the text to be synthesized, and the resultant feature vectors (observations) serve as a basis for the speech synthesis implemented by a particular filter. It should be noted that parameters related to the spectrum (or cepstrum) and the laryngeal tone parameters (*f*_0_, voicedness) are modeled separately. What is interesting in the HSS synthesis is that the models are trained on a large acoustic database before being adapted for a particular speaker. Such an approach makes it much easier to create a new synthesizer [29]. 

In 2016, Deep Mind Technologies published the findings of its study into the WaveNet system [30]. This type of speech synthesis is called parametrical synthesis. According to the authors, the system narrows the gap between the best available speech synthesis and natural speech by over 50%. Like the HSS synthesis, this method is also based on acoustic modeling. What makes it different is the elimination of the Vocoder (Voice Encoder), a coder used for analyzing and synthesizing the human speech. The audio signal is modeled directly by the same model. Because of its high computational complexity at the time of the publication, WaveNet was unable to generate a real-time speech signal, which is why this kind of synthesis is not included in this study. Later, Deep Mind went on to develop an improved model which served to create a TTS system, accessible in a virtual cloud [31]. 

VOCA devices make use of professional commercial voices, but their high quality is not the most important aspect for patients, who would rather hear their own voice. Unfortunately, the technology currently used in these systems does not allow for the provision of personalized voices [20]. Perhaps the most famous user of such a device was the British astrophysicist Stephen Hawking, who suffered from amyotrophic lateral sclerosis. Hawking used software made by the Speech Plus company. In the initial stages of the disease, he controlled the speech synthesizer with a joystick. Having lost use of his hands, he operated the device with his cheek. 

Currently, there are several companies that produce custom-made synthetic voices [32]. ModelTalker for example, a US-based company, offers to build personalized synthetic voices for the English language. The prospective user has to record between 400 and 1800 speech samples. The systems that are offered include concatenative, corpus-based and parametrical syntheses. Parametrical synthesis makes use of Deep Neural Networks (DNN). The Polish language is currently unavailable.

OKI Electric Industry Co., Ltd. in Japan employs a hybrid speech synthesizer Polluxstar to build a personalized voice that is a combination of statistical and corpus-based speech. It makes use of both acoustic units and Markov models [33].

The Google Cloud Text-to-Speech also offers a Custom Voice feature. Custom Voice allows training of a custom voice model using own studio-quality audio recordings to create a unique voice. In addition, it is possible to synthesize audio using the Cloud Text-to-Speech API. Currently, only American English (en-US), Australian English (en-AU), and American Spanish (es-US) are supported [34].

Amazon Web Services implemented a feature in Amazon Polly called Brand Voice. Amazon Polly is a service that turns text into lifelike speech, allowing one to create applications that talk and build new categories of speech-enabled products. With the Brand Voice feature, it is possible to make Neural Text-to-Speech (NTTS) voice representing your Brand’s persona. Brand Voice allows differentiating your Brand by incorporating a unique vocal identity into your products and services. There is no Polish language neural voice present [35].

Edinburgh-based CereProc is another company that offers to build synthetic voices for individual customers [36]. The technology makes use of corpus-based synthesis, and the voice building involves the adaptation of an acoustic model based on approximately four hours of recorded speech. A female voice (Pola) is available for the Polish language, but it is not possible to adjust the synthesizer to simulate one’s own voice. Acapela is another company producing custom-made synthetic voices. Again, 19 languages are available for voice banking, but Polish still is not offered. Voice Keeper is another company that supports voice banking, but it is available only for English and Hebrew. Similarly, VocalID company also supports voice banking, but only for English [37].

Microsoft Azure offers Custom Neural Voice, a set of online tools for creating voice for brands [38]. In Custom Neural Voice Pro version, 300–2000 utterances are required. Here, the Polish language is available.

In their study, Ahmad Khan et al. developed a speech synthesizer based on a patient’s voice recorded just before laryngectomy. The system of statistical speech synthesis was trained on many speakers and adapted to a 6–7min sample of the patient’s speech. Despite its low sound quality, the output resembled natural speech [20].

It is then possible to employ the existing technologies to generate high-quality speech, but it still begs the question of what quality can be obtained for a dysphonic voice.

The following study aimed to prepare speech synthesis voice for a patient with changes in the larynx, causing hoarseness, affecting perceptual judgment and the acoustic signal parameters. In addition, we checked if it is possible to generate speech quality close to the original recordings using the MUSHRA listening test. Finally, the obtained synthetic voice was compared to the voice of a professional speaker, and after comparison, the result received a higher quality relative score to the synthetic professional voice.

## 2. Materials and Methods

Back in 2014, the authors were approached by a person seeking help for someone close who had cancer. It turned out that in a few days the sick person was to undergo total laryngectomy, which would result in a loss of natural voice. At the time, it was impossible to predict the course of disease following the surgery. However, the authors were promptly engaged in a project aimed at the design of a speech synthesizer using prosody, close to natural speech. In practical terms, a task like this involves designing a corpus-based synthesizer using unit-selection speech synthesis, or one based on a statistical parametric speech synthesis system. The solution described in this paper guaranteed repeatability as well as versatility, allowing for the implementation of such projects on a larger scale.

In both types of synthesis, it was very important to build a sufficiently extensive acoustic data repository to serve as the heart of the system. An acoustic database should include a variety of acoustic units (phones, diphones, syllables) in a number of different contexts and occurrences, and of varying durations. The first stage of building an acoustic database involved creating a balanced text corpus. This required extracting from a large text database a certain number of sentences that would best meet the input criteria, for example, the minimum and maximum number of acoustic units in a sentence.

The larger the database, the more likely it was that the selected sentences will meet the set criteria. It was then important to find a balance that would ensure an optimal database size while maintaining the right proportion of acoustic units characteristic of a particular language. The speech corpus was built in a semi-automatic way and then corrected manually. Sentences selected with this method had to be manually verified in order to eliminate any markers, abbreviations and acronyms which were not expanded in the initial preprocessing. The sentences were selected by the greedy algorithm. The operation of this algorithm consists of iterative extraction of a number of sentences from a very large text set. All the sentences were also manually checked to ensure that they did not contain material that would be too hard to pronounce or contains obscene or otherwise loaded material which would introduce an emotional bias to the recordings. More information about balancing corpus is included in these articles [39,40]. 

The recordings were made in a recording studio during a number of several hours’ long sessions. Each consecutive session was preceded by a hearing of the previously recorded material in order to establish a consistent volume, timbre, manner of speaking, etc. [27,39]. 

The final stage in the construction of an acoustic database, following the recordings, was the appropriate labeling and segmentation. The segmentation of the database was carried out automatically, using statistical models, or heuristic methods, such as neural networks. Such a database should then be verified for the accuracy of the alignment of the defined boundaries of acoustic units.

### 2.1. Constructing the Corpus 

The corpus built for the recordings contained a selection of parliamentary speeches. Initially, it was a 300 MB text file containing 5,778,460 sentences. All the metadata was removed, and all the abbreviations, acronyms and numbers were replaced by full words. Then, the SAMPA phonetic alphabet was used to generate a phonetic transcription. The SAMPA phonetic is a computer-readable phonetic alphabet. A SAMPA transcription is designed to be uniquely parsable. As with the ordinary IPA, a string of SAMPA symbols does not require spaces between successive symbols.

Two algorithms of the phonetic transcription were compared: the rule-based system developed for the Festival system, and the automatic method based on decision trees. The use of decision trees proved to be far more effective, ensuring higher accuracy in the phonetic transcription [39]. The balancing of the corpus was implemented by means of a greedy algorithm. This solution best fulfilled the given input criteria such as the number of phonemes, diphones, triphones making up the length of the sentence, or the number of segments in the final corpus. For the purpose of balancing the CorpusCrt program was used, which was written by Alberto Sesma Bailador 1998 at the Polytechnic University of Catalonia and was distributed as freeware [40].

An example input sentence in our initial corpus is in its orthographic and phonetic form represented by (a) orthography, (b) phonemes, (c) diphones, and (d) triphones.

- z jakim niezrównanym poczuciem humoru opisuje pan swoją marszczącą się wątrobę
- # z j a k i m n’ e z r u v n a n I m p o tS u ts’ e m x u m o r u o p i s u j e p a n s f o j o~ m a r S tS tS o n ts o~ s’ e~ v o n t r o b e~ #
- #z zj ja ak ki im mn’ n’e ez zr ru uv vn na an nI Im mp po otS tSu uts’ ts’e em mx xu um mo or ru uo op pi is su uj je ep pa an ns sf fo oj jo~ o~m ma ar rS StS tStS tSo on nts tso~ o~s’ s’e~ e~v vo on nt tr ro ob be~ e~#
- #zj zja jak aki kim imn’ mn’e n’ez ezr zru ruv uvn vna nan anI nIm Imp mpo potS otSu tSuts’ uts’e ts’em emx mxu xum umo mor oru ruo uop opi pis isu suj uje jep epa pan ans nsf sfo foj ojo~ jo~m o~ma mar arS rStS StStS tStSo tSon onts ntso~ tso~s’ o~s’e~ s’e~v e~vo von ont ntr tro rob obe~ be~#

The parliamentary speech corpus was divided into 12 sub-corpora, 20 MB each [20]. The division was made on the grounds of the maximum corpus size that can be accepted by the Corpus CRT program.

The following criteria were applied for the selection of the most representative and balanced sentences:

- Each sentence should contain a minimum of 30 phonemes;
- Each sentence should contain a maximum of 80 phonemes;
- The output corpus should contain 2,500 sentences;
- Each phoneme should occur at least 40 times in the corpus;
- Each diphone should occur at least 4 times in the corpus;
- Each triphone should occur at least 3 times in the corpus (this particular criterion can only be met for the most frequently used triphones).

These assumptions were made on the basis of [41,42,43].

**Figure 1:**
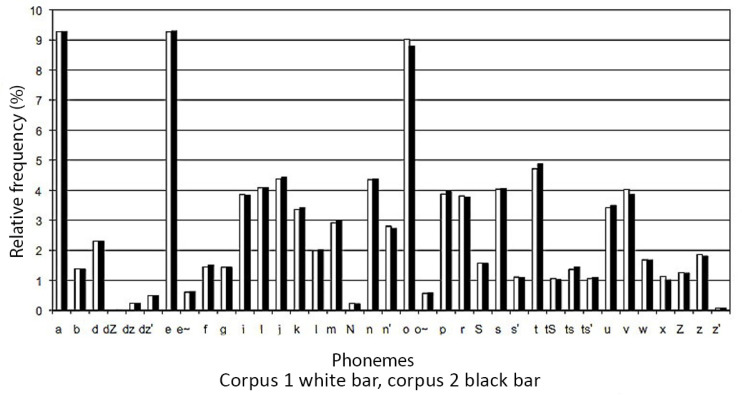
A comparison of frequency distribution of phonemes in two random parliamentary sub-corpora.

After the first balancing process, 12 different sub-corpora, each containing 2500 sentences, were created. Each sub-corpus contained approximately 189,000 phonemes. The frequencies of phonemes proved to be very similar in all of the sub-corpora. Figure 1 illustrates the percentage value of frequency distribution in two randomly selected parliamentary sub-corpora.

After the second balancing process, the total number of diphones had increased (from 148,479 to 150,814), the number of diphones occurring less than four times had decreased (from 175 to 68), and the number of different diphones had increased (from 1096 to 1196). The total number of triphones had increased (from 145,979 to 148,314), and so had the number of different triphones (from 11,524 to 13,882).

**Figure 2:**
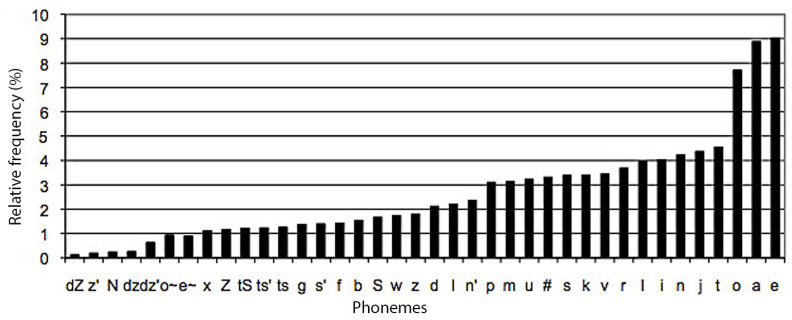
Phoneme frequency distribution in the final version of the corpus.

**Figure 3:**
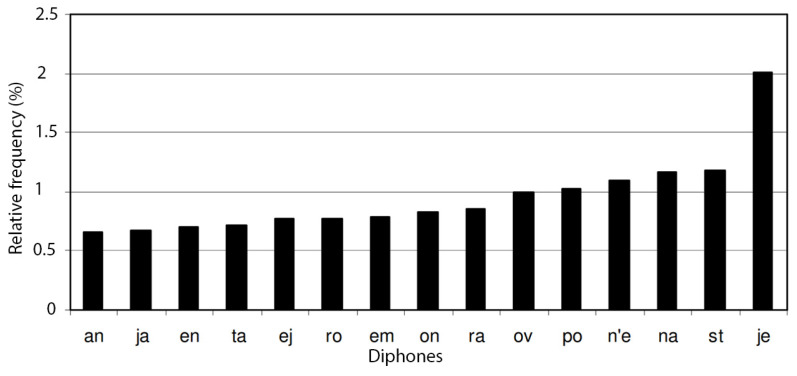
The 15 most common diphones. They account for 14.22% of all diphones in the final corpus.

**Figure 4:**
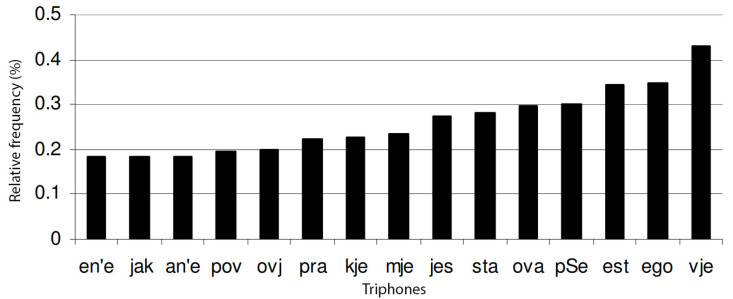
The 15 most common triphones. They account for 4.09% of all triphones in the final corpus.

The ultimate corpus contains interrogative and imperative sentences and was also supplemented with words of less frequent occurrence. The frequency distribution of particular phonemes is shown in Figure 2. The 15 most common diphones are shown in Figure 3, and the 15 most common triphones are shown in Figure 4.

The final stage of the corpus construction involved manual correction, which allowed for the elimination of sentences that were meaningless or difficult to utter. Ultimately, the corpus is made up of 2150 sentences.

In its final form, the corpus was used in a doctoral dissertation concerned with the optimization of cost function in corpus-based synthesis for the Polish language [39].

### 2.2. Recordings

Due to the patient’s condition and the time limitations resulting from the planned surgery, the recordings could not be held in a recording studio. Instead, they were made in the patient’s home. To ensure a better quality, an acoustic booth was used. The recordings were carried out with the help of EDIROL R-09HR, which was placed 60 cm from the mouth. EDIROL R-09HR is a professional, high-resolution recorder with built-in stereo condenser microphone. During the recording, a written text was displayed for the speaker and the person in charge of the recording. The acoustic database was recorded with a 48 kHz sampling frequency and a 16-bit resolution in the WAV format. Each consecutive session was preceded by an examination of the previously recorded material in order to establish a consistent intonation and manner of speaking. The first session had to be repeated as the sentences had been read too quickly. At the second attempt, the recording process was improved as the patient tried to articulate the sentences in a louder voice, and the microphone was placed closer to the speaker, i.e., 50 cm.

The entire recording was completed in two 2 h sessions, finishing, a few hours before the patient was transferred to the hospital. The whole of the corpus, consisting of 2150 sentences, was recorded. The synthetic voice was trained on 2000 sentences. 100 sentences were selected as a validation set and were used to determine the best model during after the training was completed. Finally, out of 100 sentences a set of 50 sentences was used to carry out the listening tests (MUSHRA).

The corpus containing 2000 sentences has been used in the very first unit selection speech synthesis system programmed by the authors of this paper for non-commercial use. All of the audio files used in this system have been accepted as the acoustic database of the ELRA project (http://catalog.elra.info/product_info.php?cPath=37_39&products_id=1164; http://syntezamowy.pjwstk.edu.pl/korpus.html accessed on 5 April 2022). ELRA is involved in a number of projects at the European and international levels. These projects address various issues related to Language Resources, including production, validation, and standardisation.

### 2.3. Acoustic and Auditory-Perceptual Assessment of Voice Quality 

Due to dysphonia in the patient’s voice, the RBH auditory-perceptual scale was used to assess its quality [44]. The RBH auditory-perceptual scale is used in German clinics and is recommended by the Committee on Phoniatrics of the European Laryngological Society [45]. The RBH acronym is used to denote the following features:

- R—Rauigkeit (roughness) – the degree of voice roughness deviation caused by irregular vocal fold vibrations;
- B—Behauchtheit (breathiness) – the degree of breathiness deviation caused by glottic insufficiency;
- H—Heiserkeit (hoarseness) – the degree of hoarseness deviation.

Ratings of 0, 1, 2, and 3 are used for all parameters on the RBH scale, with reference to the different degrees of vocal disorder: ‘0’ = normal voice, ‘1’ = a slight degree, ‘2’ = a medium degree, and ‘3’ = a high degree.

The perceptual voice assessment was performed by two independent specialists who had completed an RBH training program and had extensive experience in voice signal evaluation. The experts were trained at a university. The training process was divided into three stages; each stage lasted 28 h. After each step, an exam checked the quality of annotation. Upon successfully finishing the training, another learning process was introduced with RBH Learning and Practice mobile application. The experts had been working for three years with an annotation of the speech signal. 

The assessment showed dynamic voice changes throughout the recordings, with R = 0, B = 1, H = 0 at the beginning of the recordings, and R = 1, B = 1, H = 1 at the end. These ratings indicated dysphonic changes in voice quality, pointing to dynamic changes taking place during the recordings.

To better illustrate the changes, an acoustical analysis using AVQI (v. 02.03) was carried out (The Acoustic Voice Quality Index) [45,46]. The Acoustic Voice Quality Index is a relatively new clinical method used to quantify dysphonia severity. The method is calculated on the basis of a signal from a sustained vowel and samples of speech. To determine its value, a weighted combination of 6 parameters is taken into account: shimmer local, shimmer local dB, harmonics-to-noise ratio (HNR), general slope of the spectrum and tilt of the regression line through the spectrum and smoothed cepstral peak prominences (CPPs). 

The AVQI score obtained for the patient with laryngeal cancer was 5.62, which indicates largely altered voice quality. AVQI values range from 0 to 10.

**Figure 5:**
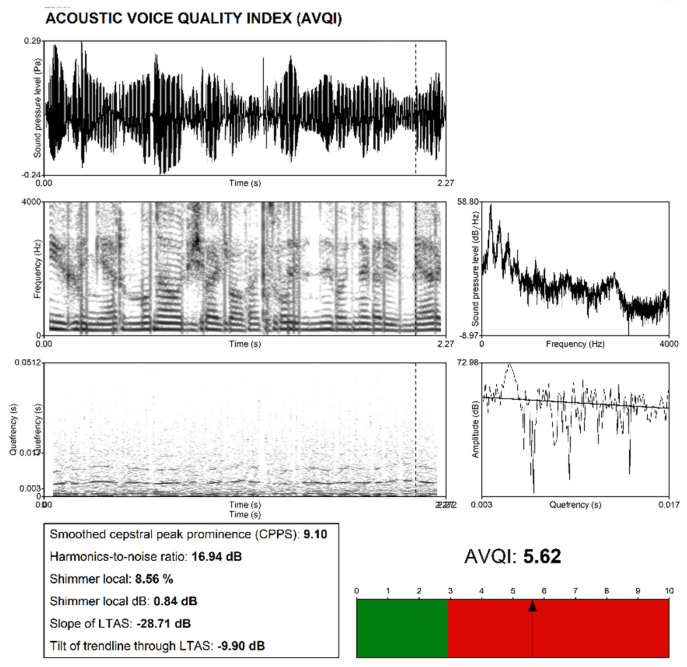
Acoustic assessment of the patient’s voice.

It was assumed that scores ≤ 3 indicate a normal, unchanged voice [45]. The patient’s voice was compared with that of a professional speaker recorded for the corpus-based speech synthesis, both using the same sentences. In order to select the professional speaker, voice samples from 30 voice talents were collected and then assessed by 8 voice analysis experts. Ultimately, the experts chose a female voice. The recordings, which were conducted in the recording studio of the Polish-Japanese Academy of Information Technology, were performed with an Audio-Technica AT2020 microphone with a pop filter, 30 cm from the microphone. The signal was recorded in the AIFF format with a 48 kHz sampling frequency and a 24-bit resolution, using the audio Focusrite Scarlett 2i4 interface. The corpus was recorded during 15 two-hour sessions, with each prompt being recorded as a separate file. After each session, the files were exported in the WAV format with file names corresponding to the prompt numbers in the corpus. The recordings were then checked for distortions and external noises, as well as for mistakes made by the speaker. A total of 480 prompts were re-recorded [27]. The values obtained for the voice were: AVQI = 1.61, and R = 0, B = 0, H = 0 on the perceptual scale. Figure 5 shows a graph with the acoustical analysis using AVQI calculated for the patient.

### 2.4. Segmentation of Audio File 

The next step, after recordings, was an automatic segmentation of the corpus. This was carried out by means of a program based on the Kaldi project [47]. Kaldi is an open-source speech recognition toolkit, written in C++. The segmentation was performed using a technique called ‘forced alignment’, which involves matching phone boundaries on the basis of a file containing phonetic transcription. First, the program created an FST graph whose states correspond to the consecutive segmental phonemes of the analyzed phrase. The phonetic transcription for the segmentation was prepared on the basis of an orthographic transcription using a Polish language dictionary with SAMPA transcriptions. Foreign words and proper nouns were transcribed manually.

### 2.5. Creating Synthetic Voice

The authors set out to create a new voice using the Merlin library [48], a toolkit for building statistical parametric speech synthesis by means of Deep Neural Network. This approach must be used in combination with metasystem Festival, responsible for implementing phonetic transcription, linguistic features and the World library as a vocoder [49]. The World library also provides tools for analysis, processing and recording. In Festival, the following features were calculated:

- Context dependent phones (previous phoneme, next phoneme);
- Syllable structure (current, previous and next syllable);
- For each of syllable (stress accent and length of syllable);
- Position phoneme in syllable;
- Position phoneme in phrase;
- Position of stressed syllable in phrase.

The first step was to define acoustic parameters based on the recordings. This involved calculating the values of fundamental frequency (*f*_0_), voicing levels, mel-generalized cepstral coefficients (MGCC) [28], and band aperiodicity, which expresses the value of the aperiodic energy signal. Each of the parameters were normalized to the mean value of 0, and their variance value equaled 1. All the parameter values for a given frame constitute its vector of acoustic properties. For *f*_0_ only values corresponding to the voiced signal frame was used, for non-voiced frames value 0 was used.

Additionally, the delta and delta–delta were calculated for the F0 and MGCC parameters. Thus, the F0 for every signal frame is represented by three values. Each of the MGCC parameters is defined by 60 parameters representing the amount of energy for each sub-band. Ultimately, together with the delta and delta–delta, each signal frame is represented by 180 values. 

Once the sentence to be synthesized has been entered, the acoustic model predicts the acoustic parameter values using the obtained linguistic parameters. The latter were extracted at the phoneme level, while the acoustic parameters were extracted at the frame level. Their numbers differ, which makes model training difficult. In order to resolve the problem, information about the boundaries of phoneme states obtained in the segmentation process was used. Each state was matched with corresponding frames. The vector representing linguistic properties of a given state was copied a required number of times, and an index was added to it. Data prepared in this way contained for each frame its vector of linguistic properties and the corresponding vector of acoustic parameters. This represents, respectively, the input and the desired output required to train an acoustic model.

However, the information about the states is not available during the synthesis process. For this reason, a model that will predict their duration on the basis of their linguistic parameters needs to be developed. The acoustic models and phoneme duration models were trained using Python Theano library [50]. The Theano library is integrated with Merlin and contains implemented statistical models based on deep neural networks. In addition, this allows for a very fast computation of mathematical expressions by using specialized GPUs.

## 3. Results

### 3.1. Experiments

**Figure 6:**
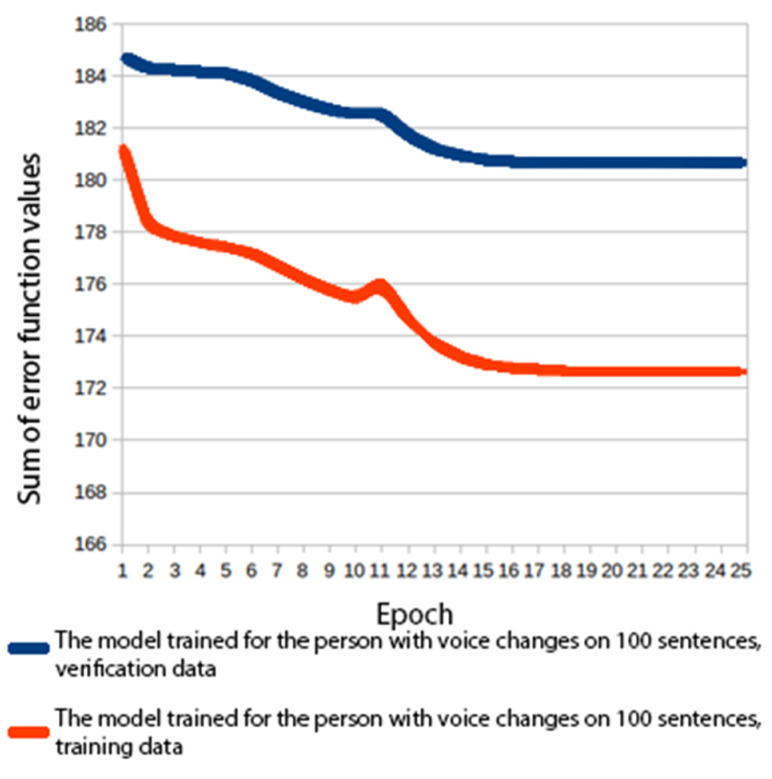
Error function values for a voice trained on 100 sentences.

**Figure 7:**
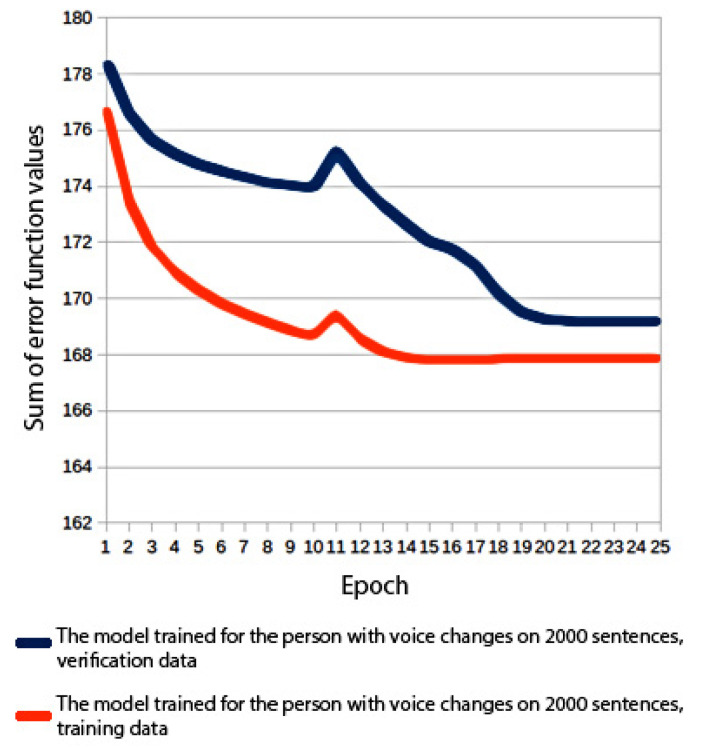
Error function values for a voice trained on 2000 sentences.

**Figure 8:**
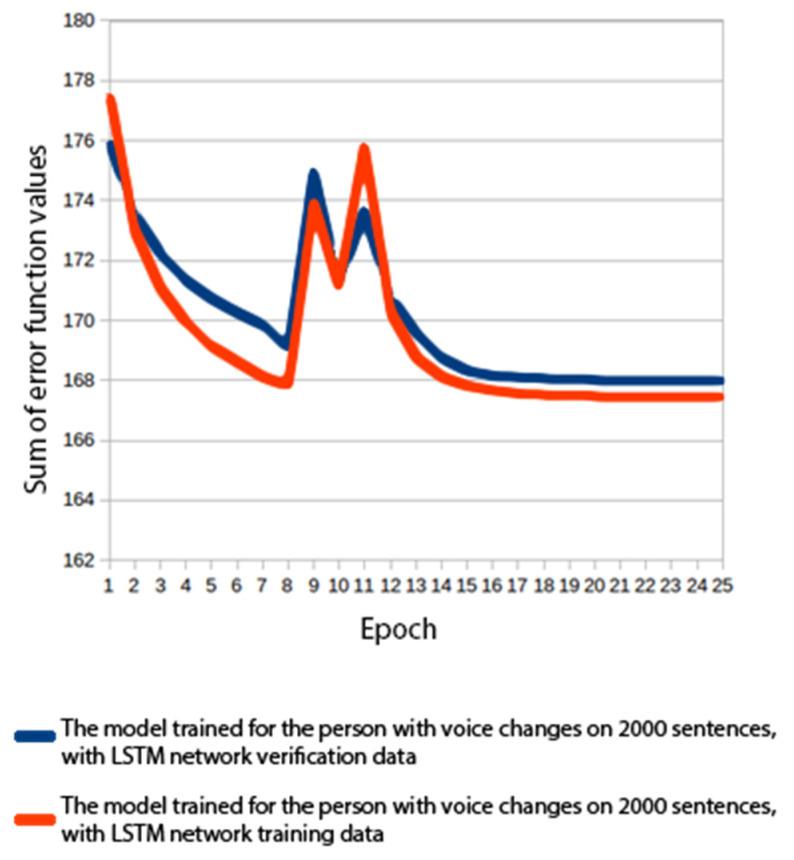
Error function values for a voice trained on 2000 sentences using LSTM layers.

**Figure 9:**
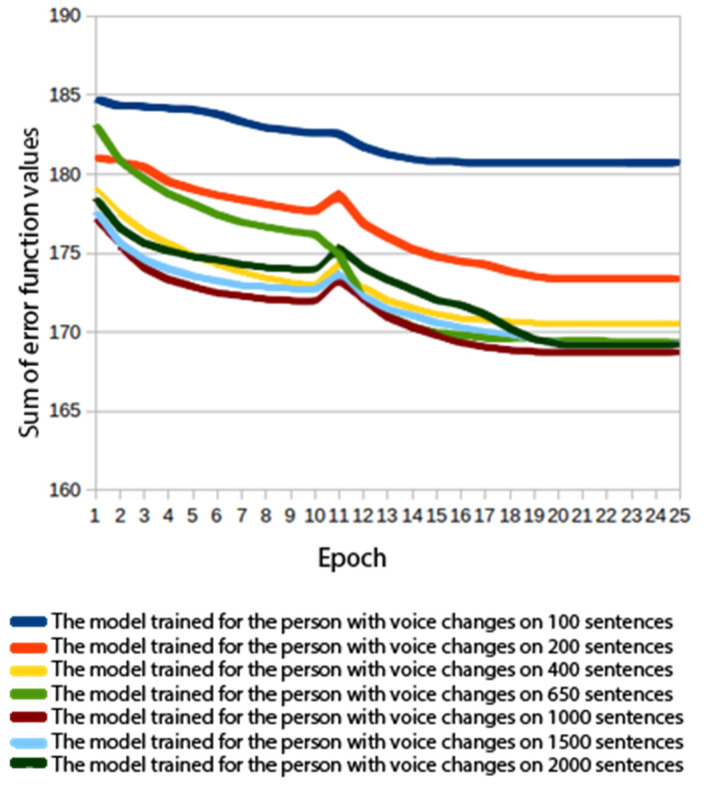
Error function values for a varying number of sentences.

A number of experiments were carried out where voices were built with varying amounts of training data and different acoustic model architectures. In order to compare the models, values of the error function calculated for the verification data were used. The verification data constituted 10% of the training set. The mean squared error was used in the process. The values shown in Figure 6, Figure 7, Figure 8 and Figure 9 are the MSE sum for 180 mel-generalized cepstral coefficients, 3 parameters describing the fundamental frequency (*f*_0_) and 3 parameters describing the aperiodic band. The parameters were normalized to the mean value of 0 and the variance of 1. In all experiments, the models were trained for 25 epochs and a model from the best performing epoch was used.

#### 3.1.1. Experiment 1: Building a Voice with 100 Sentences

The first model was used to verify the system, so it was trained on a small number of sentences. 100 sentences were randomly selected from the corpus, of which 90 were used to train the models (training data). The remaining 10 sentences were used for verification purposes (verification data). A multilayer perceptron was used for the acoustic modelling. It consisted of an input layer (1), hidden layers (2) and an output layer (3). There were 6 hidden layers, each consisting of 1024 neurons. The hyperbolic tangent was chosen to act as the activation function. An identical neural network was employed for the modelling of phoneme state durations. 

In both cases, computations were performed without a GPU. They were made on a computer with an 8-core processor Intel Core i7-4790 3.60 GHz, 16 GB RAM. As Theano performs automatic data-parallel computations, all of the processor cores were utilized. The speech generated by the resultant models was comprehensible, though not very natural sounding. However, the experiment helped verify the correct functioning of the system.

#### 3.1.2. Experiment 2: Building a Voice with 2000 Sentences

The training data set and the verification set consisted of 2000 and 100 sentences, respectively. Both models were trained with the same neural network architecture as in the first experiment. In both cases, computations were performed with a CPU only. The resultant models made it possible to generate speech that sounded noticeably more natural than the speech generated in experiment 1. Figure 6 and Figure 7 show a graph of the error function during the voice training stage. The problem of overfitting was significantly reduced compared to the model trained with 100 sentences. 

#### 3.1.3. Experiment 3: Building a Voice with an Acoustic Model Based on a Recurrent Network 

This experiment was carried out with the same data set as in experiment 2. What made it different was an altered architecture of the acoustic model neural network (the model of the phoneme state durations remained unchanged). The last two layers of the perceptron were replaced with two LSTM layers [51,52]. The LSTM layer was recurrent, which means that the value predicted for the prior sample was at once the input value for the current sample. Thanks to this property, neural networks containing LSTM layers were used for sequence modelling. 

Apart from a perceptron with a hyperbolic tangent, a single LSTM block contained three perceptrons with a sigmoid activation function. The first of these was a forget gate, designed to discard any unimportant information from prior elements of the sequence. Next was the input gate, which filtered information in the current element. The third gate was the output gate, which decided which information should be passed to the subsequent elements of the sequence. Each of the LSTM layers consisted of 384 blocks. Computations performed in a single LSTM block were more complex than those in the perceptron. The time needed to train the model with a processor was estimated at 500 h. Therefore, it was decided that a GPU would be used. The GPU processor (Nvidia GTX 760) made it possible to train the model in 31 h and 27 min. 

Figure 8 shows a graph of the error function values during the model training. The application of LSTM layers practically eliminated the problem of overfitting.

#### 3.1.4. Experiment 3: Building a Voice for 100, 200, 400, 650, 1000 and 1500 Sentences

In order to investigate the effect of data volume on voice quality, additional acoustic models were built for 200, 400, 650, 1000 and 1500 sentences, respectively [39]. Figure 9 shows a graph of the error function values for a varying number of sentences. There was a striking difference between 100 and 200 sentences. There was also a noticeable leap between 200 and 400 sentences. A further increase in the number of sentences used did not affect the rate at which the error function values fell.

The experiments discussed above led to the construction of 3 synthetic voices: two for 100 and 2000 sentences using an MLP network, and a third voice built on the basis of 2000 recordings using a recurrent network with LSTM layers. The voices built in experiment 4 were designed to examine the impact of different amounts of data on the quality of the models and were excluded from further evaluation.

### 3.2. MUSHRA

The listening tests were conducted using the MUSHRA methodology. In a MUSHRA test, the listener is presented with a professional voice-over talent recording as the reference, (so called proper reference) and samples of generated speech to be evaluated. These generated systems include a so-called anchor. In addition, one of the systems served as a hidden reference. The hidden reference used in our tests is the same a voice-over talent recording as it was used as the proper reference. Such an approach made it possible to verify that the listeners assessed the systems against the reference. An anchor was required to be perceived as inferior in quality to the hidden reference.

The tests were carried out by means of webMUSHRA. A total of 25 expert listeners participated in the tests, each of whom assessed 10 sentences in one test. The listeners were instructed to first listen to the reference recording and then assess each system on a 0–100 numerical scale. The results are shown in Table 1. The sentences used in the test came from a specially designed test corpus also named validation corpus and were not used for training or verification purposes. The purpose of creating the corpus was to obtain a set of sentences that would meet specific requirements different from those used to develop the main corpus [53]. It was decided to get a small corpus and, at the same time, the biggest possible coverage of different acoustic units, different from the ones included in the acoustic database. The variety of corpora was supposed to ensure the naturalness and comprehensibility of generated phrases occasionally occurring in the main corpus. The test corpus was prepared in the CorpusCrt application [40]. Sentences were compiled from three different linguistic bases, containing texts from newspapers on various subjects. Before the test corpus was created, it was required to generate the phonetic transcription for phonemes diphones and triphones for the whole database. It was decided to limit the size of the test corpus to 100 short statements (max. 60 phonemes in each sentence). The criteria of the sentence selection referred to their maximum length, the number of occurrences of various acoustic units, and different phoneme configurations. During corpus balancing, it was decided that each phoneme should occur at least 25 times, each diphone and triphone should occur at least once. Because of the small size of the corpus, obtaining all the diphones and triphones was impossible; however, the necessary condition ensured a variety of occurrences of mentioned acoustical units.

The results obtained in the tests indicated a very high quality of the synthetic voice of the patient (Table 1). A difference of 0.05 in relative score in favor of the best patient’s synthetic voice 3 LSTM compared to the best professional synthetic voice accounted for by a better adjustment of the acoustic parameters (Table 1). The obtained results indicated that the best synthetic patient voice is more matched to the original patient recordings than the professional synthetic voice concerning the professional recordings.

As voice 3 required the use of GPU, systems 2 and 3 were compared to see if their ratings were different. The ratings of both systems had an equal modal distribution and equal variances. P value = 0.651 and t = 0.452 indicated that the ratings of the two systems are not statistically different. This ultimately led to a decision to transfer the system to a virtual speech synthesizer, which would benefit the patient. Due to the computational complexity of the LSTM layer, the model trained with LSTM was too slow to be used on a computer without a GPU. For this reason, it was not placed on the virtual machine that was presented to the non-professional voice. 

#### The Analysis of Acoustic Parameters Errors

Table 2 shows the values of acoustic parameters errors calculated on the basis of test sentences generated by means of 3 trained systems. The following acoustic parameters were applied:

- MCD—Mel-Cepstral Distortion;
- BAPD—band aperiodicity distortion;
- F0-RMS represents the root mean square of deviations in fundamental frequency values;
- F0-correlation, the value of Pearson’s correlation coefficient for the fundamental frequency;
- VUV (voiced-unvoiced error rate) indicates the percentage of incorrect predictions of voicedness [48].

The analysis of the data shown in Table 2 indicated that voice 2, i.e., a voice for the cancer patient, had the best BAPD value as well as having the highest correlation between F0 and the recording. On the other hand, the best values of MCD, F0-RMS and VUV were obtained for the LSTM-trained voice. The professional voice had better MCD and VUV% values while its BAPD value was equal to that of the cancer patient. The parameter values of the fundamental tone deviation (F0-RMS) and the fundamental frequency F0-correlation proved to be worse in the professional voice. 

In order to enable researchers to repeat or modify the conducted experiments, a GIT repository was created. The repository contains the Merlin Repository with all the modifications and scripts. The Supplementary Materials contain recordings of voice talent, patient voice, and synthetic voice of voice talent and patient.

The synthetic voice was made available to the patient in the form of a virtual machine in the VirtualBox13 environment. The text is synthesized with a single command at the terminal level. The synthesizer works fast in Linux. However, transferring it to a virtual machine affects its operating speed.

## 4. Discussion and Conclusions

The study aimed to prepare speech synthesis voice with changes in the larynx, causing hoarseness and affecting perceptual and acoustic signal parameters. The quality for the person with voice changes obtained a relative score of 0.71 for MLP and LSTM, where the relative score is defined as a recording’s mean value divided by synthetic voice mean. Interestingly, a higher voice quality than professional voice was achieved, where the relative score equals 0.66. MUSHRA results of patient MLP voice trained on 2000 sentences obtained 69.40 compared to 63.63 for professional voice. Creating such a voice was possible, but perceptual differences indicated that the patient voice sounded better than the professional voice.

According to their study, Repova et al. [21], 61 patients were scheduled for total laryngectomy for T3-T4a laryngeal or hypopharyngeal cancer with uni- or bilateral neck dissection the regional lymph node involvement. A total of 31 patients were assessed as unsuitable for voice recordings due to low voice quality before surgery or unsatisfactory cooperation and compliance. Of the remaining 30 patients, 18 were willing and able to complete voice recordings. Of the 18 patients, 11 patients had a voice prosthesis implanted. Each patient recorded between 210 and 1400 sentences. For most, unit selection (US) or hidden Markov model (HMM) systems were used to perform personalized speech synthesis. However, the quality of speech synthesis was not evaluated. Overall, only 7 patients eventually began using TTS technology in the early postoperative period. However, the frequency and total time of use were significantly better in the first postoperative week than later in the hospital stay, when the device’s effort gradually decreased. Finally, 6 patients are actively using the software. One of these patients was a lecturer. The frequency and total device use time were significantly better in the first postoperative week than later in the hospital stay, when the effort to use the device gradually decreased. The gold standard for voice rehabilitation after total laryngectomy is tracheoesophageal speech with the voice prosthesis placement. The disadvantage of this approach is the necessity of regular replacement of the voice prosthesis due to the device’s lifetime. In their study, Repova et al. [21] obtained results that indicate that voice banking and speech synthesis can be an opportunity to increase the quality of life.

Statistical speech synthesis created by recording complete corpus allows the generation of more natural-sounding speech than that obtained by adapting acoustic models to a particular patient, as reported in Ahmad Khan et al. [20]. The authors used the system of statistical speech synthesis was that trained on many speakers and adapted on a 6–7-min’ sample of the patient’s speech. Despite its low sound quality, the output resembled natural speech. 

The created corpus in this study is representative of the Polish language. It enables high-quality, corpus-based and HSS speech synthesis. The signal segmentation methods developed in the study ensure a high degree of accuracy, as confirmed by the author’s previous studies [27,39,41,54]. This work is innovative for the Polish language. 

The method developed in the course of the study makes it possible to create a new synthetic voice for the Polish language by means of a statistical parametric speech synthesis system. Despite significant changes in the patient’s voice, reflected in the RBH scale features and the AVQI parameters, the results obtained in the study were very promising, as confirmed by the MUSHRA test. As a result, this method can be employed to develop a synthetic voice for a person awaiting total laryngectomy, allowing them to speak with their own voice, which ensures the patient’s better mental wellbeing.

Having been presented with the speech synthesizer, the total laryngectomy patient was clearly moved being able to hear his own voice and expressed full approval of the quality of the synthesis.

## Figures and Tables

**Table 1:** MUSHRA test results.

	Patient’s Voice	Patient’s Synthetic Voice 1 MLP	Patient’s Synthetic Voice 2 MLP	Patient’s Synthetic Voice 3 LSTM	Professional Voice	Professional Synthetic Voice MLP
Relative score *	-	0.36	0.71	0.71	-	0.66
Mean value	97.62	35.48	69.40	69.74	96.21	63.63
Median	100	34	71	72	100	66
STD	5.54	21.52	19.42	18.35	8.01	19.41

* Relative score = recording’s mean value/synthetic voice mean value.

**Table 2:** Values of acoustic parameter errors calculated for verification data.

Voice ID	MCD (dB)	BAPD (dB)	F0-RMS (Hz)	F0-Correlation	VUV %
Voice 1	5.489	0.142	29.787	0.489	11.792
Voice 2	4.779 *	**0.133**	26.096*	0.635 *	9.059*
Voice 3 LSTM	**4.731**	0.134	**25.438**	0.629	**8.308**
Professional voice	4.186	0.133	31.116	0.558	5.689

* Statistically significant in comparison to professional voice, *p*-value <0.05. All differences between acoustic parameters except the BAPD (dB) parameter for voice 2 and professional voice are statistically significant. Between voice 2 and voice 3, only the VUV % parameter is statistically significant (*p*-value <0.05). The bold font is used to indicate the best acoustic parameter among all synthetic voices.

## Data Availability

The GIT repository can be accessed at https://github.com/kubapb/merlin, (accessed on 5 April 2022).

## Supplementary Materials

The following supporting information can be downloaded at: https://www.mdpi.com/article/10.3390/s22093188/s1. The materials contain recordings of voice talent, patient voice, and synthetic voice of voice talent and patient.
